# Explaining and predicting the increased thorax injury in aged females: age and subject-specific thorax geometry coupled with improved bone constitutive models and age-specific material properties evaluated in side impact conditions

**DOI:** 10.3389/fpubh.2024.1336518

**Published:** 2024-03-11

**Authors:** Miguel A. Corrales, John Henry Bolte, Bengt Pipkorn, Craig Markusic, Duane S. Cronin

**Affiliations:** ^1^Department of MME, University of Waterloo, Waterloo, ON, Canada; ^2^Injury Biomechanics Research Center, Ohio State University, Columbus, OH, United States; ^3^Division of Vehicle Safety, Department of Mechanics and Maritime Sciences, Chalmers University of Technology, Gothenburg, Sweden; ^4^Autoliv Research, Vårgårda, Sweden; ^5^Honda Development & Manufacturing of America, Raymond, OH, United States

**Keywords:** aged population, rib fracture, thorax injury, side impact, human body model (HBM), bone constitutive modeling

## Abstract

Predicting and understanding thorax injury is fundamental for the assessment and development of safety systems to mitigate injury risk to the increasing and vulnerable aged population. While computational human models have contributed to the understanding of injury biomechanics, contemporary human body models have struggled to predict rib fractures and explain the increased incidence of injury in the aged population. The present study enhanced young and aged human body models (HBMs) by integrating a biofidelic cortical bone constitutive model and population-based bone material properties. The HBMs were evaluated using side impact sled tests assessed using chest compression and number of rib fractures. The increase in thoracic kyphosis and the associated change in rib angle with increasing age, led to increased rib torsional moment increasing the rib shear stress. Coupled with and improved cortical bone constitutive model and aged material properties, the higher resulting shear stress led to an increased number of rib fractures in the aged model. The importance of shear stress resulting from torsional load was further investigated using an isolated rib model. In contrast, HBM chest compression, a common thorax injury-associated metric, was insensitive to the aging factors studied. This study proposes an explanation for the increased incidence of thorax injury with increasing age reported in epidemiological data, and provides an enhanced understanding of human rib mechanics that will benefit assessment and design of future safety systems.

## 1 Introduction

The aged adult population have demonstrated a higher mortality compared to the younger adult population (1), and the female population have a higher incidence of injury compared to the male population for similar car-crash scenarios (2). Amongst the aged population, thorax injury is the leading cause of fatalities, more so for the female population (1, 3). Importantly, female aged subjects have demonstrated a large increase in severe thorax injury for side impacts when compared to younger counterparts (3). Thorax injury has been correlated with thorax deformation (4) and rib fracture of varying severities (5). The thoracic cage load bearing tissues are the ribs, costal cartilage and sternum. It has been suggested that changes in material properties and geometry with increasing age may potentially contribute to the increased incidence of thorax injury and morbidity in the older adult population (6–8). Particular to the ribs, the cortical material properties have been measured in coupon tension testing (8). It was shown that the Young's modulus, strength (ultimate stress) and the strain to failure decreased with increasing age. Importantly, cortical bone demonstrates anisotropic behavior in coupon testing experiments with the primary direction oriented along the osteon direction (8).

Finite element human body models (HBMs) serve as crucial tools for evaluating human response and injury risk in vehicle crashes (9). However, these models are often developed for specific anthropometric, age, and sex requirements. The Global Human Body Models Consortium introduced the F05-O v5.1 detailed model (F05-O), representing a small female based on subject-specific geometry of a 26-year-old volunteer (10). The geometry was obtained through magnetic resonance imaging (MRI) and computer tomography (CT) scans of a subject reflecting the characteristics of a 5^th^ percentile female in terms of mass, stature, and BMI. Notably, the F05-O model was developed with mesh quality requirements to ensure numerical accuracy (11). The F05-O has undergone validation in various body regions, including the thorax, with specific focus on chest compression and the incorporation of hard tissue failure using element erosion (12, 13). The material models for trabecular and cortical bone in the GHBMC models are isotropic with elastic-plastic response, and simulate material failure with an element erosion criterion, based on maximum principal strain threshold.

To assess the risk of thorax injury, it is important that HBM rib models predict force-displacement response, force and displacement at fracture, and fracture location in dynamic compression anterior-posterior (A-P) loading, when compared to experimental data (14). Therefore, there have been various efforts to improve rib response in compression A-P loading. A past study suggested that subject-specific rib models, based on high-resolution CT scans implemented in high density finite element mesh, could better predict rib fracture location in anterior-posterior bending, given an adequate thickness of the rib cortical cortex (15). However, such complex and computationally expensive models can be challenging to integrate in full body HBMs. A recent study (16) successfully modified a contemporary HBM rib model to better predict force-displacement response, force and displacement at fracture, and fracture location, closely matching experimental results. The improved predictions were achieved by incorporating improved biofidelic cross-sectional geometry and anisotropic and asymmetric material models for the rib cortical and trabecular bone. Although the effect was not investigated at a full body level, the study achieved improved prediction of rib fracture and rib kinetics in an isolated rib model than can be integrated directly into full body models.

There have been various efforts to develop aged thorax models. Schoell et al. (17) specifically focused on developing a 70-year-old (YO) 50^th^ percentile male thorax model by incorporating target geometry for the ribs and sternum based on population data; however, the thoracic kyphosis remained constant. The GHBMC M50 v4.2 detailed thorax model was combined with simplified adjacent body regions, and the authors concluded that the modest geometry changes did not modify the response, while the material properties were reported to account for the changes observed between young and aged models. On the other hand, personalized full-body models have been created using global subject metrics such as mass, stature, and posture as morphing targets. These models have been used to evaluate restraint systems (18). Although these morphing targets were subject-specific, they only considered external measurements, and thus the internal geometry was not personalized. More recently, age-targeted HBM models were developed (19) by modestly modifying the rib geometry and modifying the material properties of an elastic-plastic cortical bone material model to represent the aged population. The models showed increased fracture probability with increasing age under equivalent impacts. The models were evaluated using age-adjusted injury risk curves based on cortical bone maximum principal strain. The increased injury risk found on the aged models was attributed to the changes in material properties alone and the use of age-adjusted injury risk curves, while the geometry had no effect. Recently, using a hybrid Morphing-CAD methodology, the geometry of a 26-year-old 5^th^ percentile female model (F05-O) was morphed and repostured into an average 75-year-old (YO) and a subject-specific 86YO HBM (F05-86G) (7). This approach targeted specific bone positioning and successfully achieved precise morphing targets without compromising the mesh quality. In a simple side sled impact test, the three HBMs exhibited comparable kinematic responses but displayed distinct rib fracture patterns, with the aged models demonstrating higher number of rib fractures. However, the study did not consider changes in material properties and used elastic-plastic isotropic cortical and trabecular bone models.

A recently developed cortical bone fracture model (CFraC) (20) was proposed based on bone continuum damage mechanics with a stress triaxiality fracture criterion. The cortical bone material model and fracture criterion effectively predicted fracture initiation, location, and pattern in whole bone and specimen level tests, demonstrating consistency with the experimental data and within the population variability. Importantly, the model remained functional, accurate, and numerically stable when utilizing coarse mesh sizes commonly employed in contemporary HBMs. The CFraC model has been previously implemented in the GHBMC models in isolated rib testing (16). It was concluded that including the CFraC model greatly enhanced the model's capability to predict the force and displacement at failure and fracture location.

Two human models having stature and BMI within the 5^th^ percentile female population were investigated in this study. The geometry for each model was subject specific. The 5^th^ percentile female population group was investigated in this study due to the higher incidence of injury and fatality reported for small stature females compared to average stature males (1, 2, 21, 22). The young model was the GHBMC 5^th^ percentile female model, being a subject-specific scan of a 26 YO female (10). Importantly, in the context of this study, the rib angles of the GHBMC 5^th^ percentile female young model were close to the population values. In a recent study, the young model was morphed to an 84YO subject-specific geometry (7), and that aged geometry was used in the current study. The resulting rib angles for the aged geometry were higher than the population average for 84YO subjects; however, it is reported that geometric variability increases with increasing age (23).

**Figure 1:**
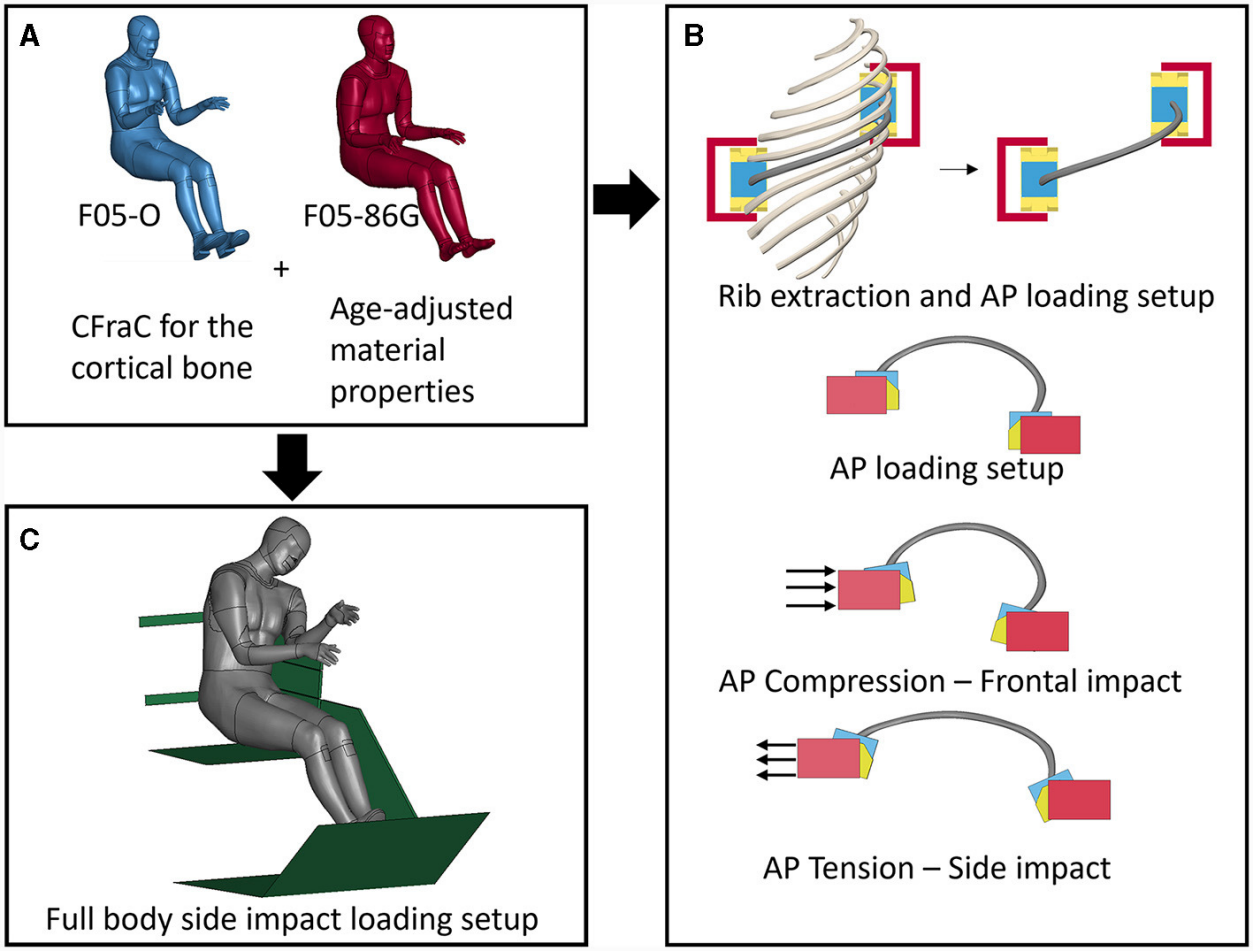
Schematic showing the young (F05-O) and aged (F05-86G) HBMs coupled with **(A)** enhanced constitutive models for the rib cortical bone, **(B)** the 6^th^ rib extraction and simulation in A-P loading, maintaining the body positioning, and **(C)** full body side-impact sled simulations.

In this study, the F05-O and the previously developed subject-specific 86-year-old small female geometry (F05-86G) model were enhanced with a recently developed cortical bone material model (Figure 1A). Then, the F05-O and the F05-86G models were enhanced with age adjusted material properties for cortical bone (F05_26_ and F05_86_). The effects of geometry and material properties were investigated separately. The 6th rib was extracted from the F05-O and F05-86G models, and loaded using previously published boundary conditions (16, 24), but in a newly-proposed A-P configuration that represented the natural anatomic position of the rib (Figure 1B). The modified rib orientation allowed for the examination of geometric changes and altered posture (i.e., increased thoracic kyphosis) with increasing age using a simplified boundary condition. The extracted 6^th^ rib was loaded in anterior-posterior tension and compression (Figure 5) to mimic side and frontal impact rib deformation, respectively, on an isolated rib. In a side impact condition, the rib span increases (tension) due to side thorax compression. In a frontal impact condition, the rib span decreases (compression) due to the frontal thorax compression. Finally, the age-adjusted full body models were simulated in a side impact sled model (Figure 1C) and assessed based on thorax compression and predicted rib fracture.

## 2 Materials and methods

First, the F05-O and a previously developed subject-specific 86-year-old small stature female model (F05-86G) were enhanced by including recently developed material models for rib cortical bone (CFraC) (20). The CFraC model was populated with age dependent material properties from tension rib cortical bone coupon testing (8). The combination of the F05-O, F05-86G and the age dependent material properties form the various models investigated in this study (Table 1). The HBMs were evaluated in a side impact sled test (9) using chest compression as a global metric for injury assessment. In addition, the rib fracture and kinetics along the 6^th^ rib were calculated and reported. The results of the young models were compared to those of the aged model and a factor analysis including the geometry and material properties was undertaken.

**Table 1:** Description of the geometry, material property, and constitutive model combinations investigated for the cortical bone.

**Model**	**Description**	**Cortical bone material model**
F05-O	Baseline GHBMC F05-O v5.1	Elastic-Plastic
F05_26_	Young geometry with young material properties	CFraC
F05_26GAmp_	Young geometry with aged material properties	CFraC
F05_86GYmp_	Aged geometry with young material properties	CFraC
F05_86_	Aged geometry with aged material properties	CFraC

### 2.1 Enhancing the GHBMC rib fracture prediction with an age dependent cortical bone continuum damage mechanics model

**Figure 2:**
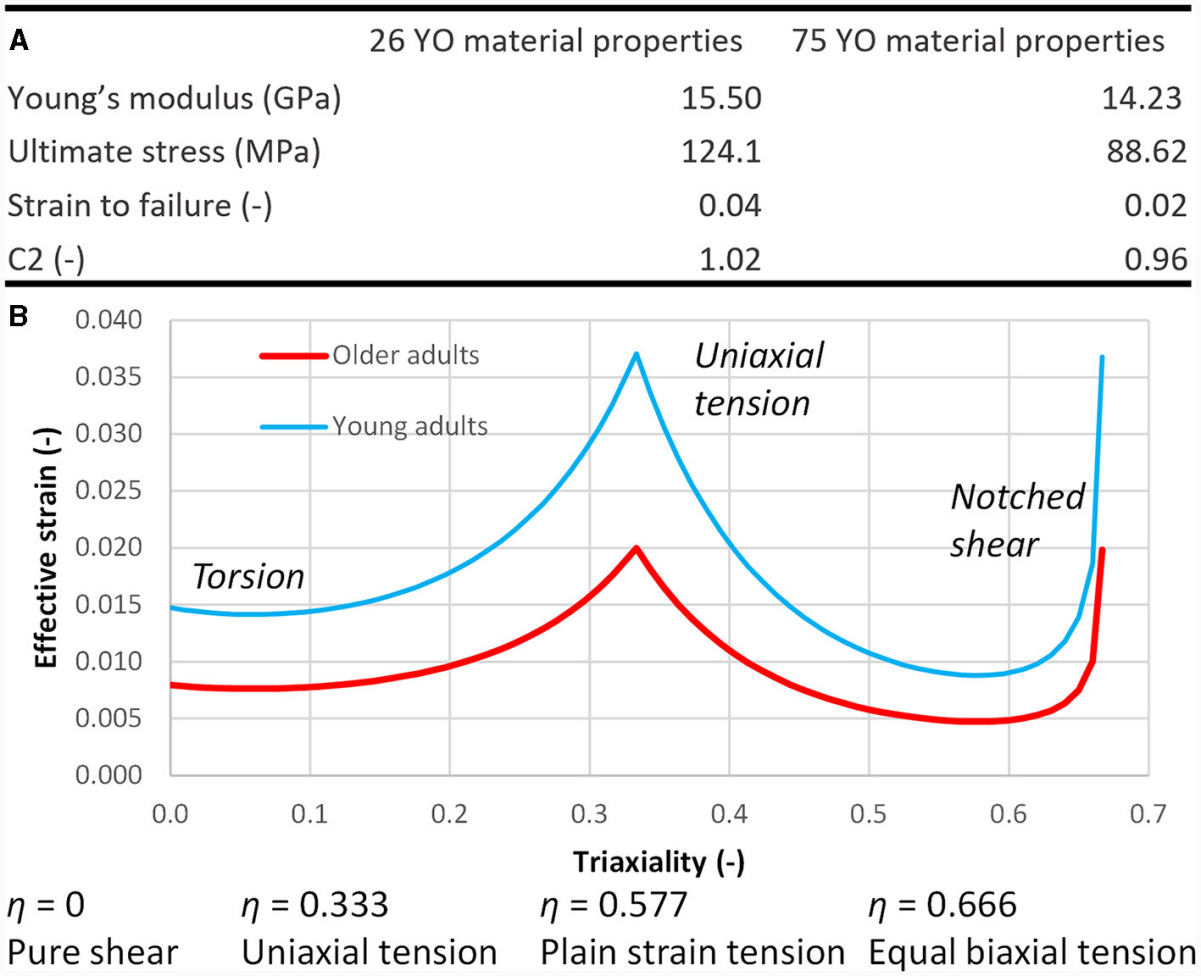
**(A)** Age adjusted material properties based on population experimental data (8). **(B)** Age adjusted effective strain-stress triaxiality curves for the CFraC constitutive model.

The enhancement of the cortical bone material model included non-isotropic properties, meaning that the primary direction of the finite element mesh of the cortical bone (2D elements) had to be oriented along the length of the rib to match the osteon direction (8). The recently developed CFraC (20) model was used to replace the isotropic elastic-plastic material model in the F05-O. The originally published material parameters of the CFraC model were based on femur cortical bone data. In this study, the Young's modulus, ultimate stress, and strain to failure material parameters were updated using rib cortical bone coupon tension testing data (8) and implemented in the CFraC model (Figure 2).

A set of parameters for the CFraC material model to represent the older adult population was developed (Figure 2A). The effective strain vs. stress triaxiality failure criterion curve was scaled based on the strain to failure of rib cortical bone from young and aged subjects under uniaxial tension coupon testing (Figure 2B). The CFraC model parameter “C2” was modified to match the strain to failure of cortical bone coupon testing under uniaxial tension (Figure 2B).

### 2.2 Spine and local rib geometric assessment

**Figure 3:**
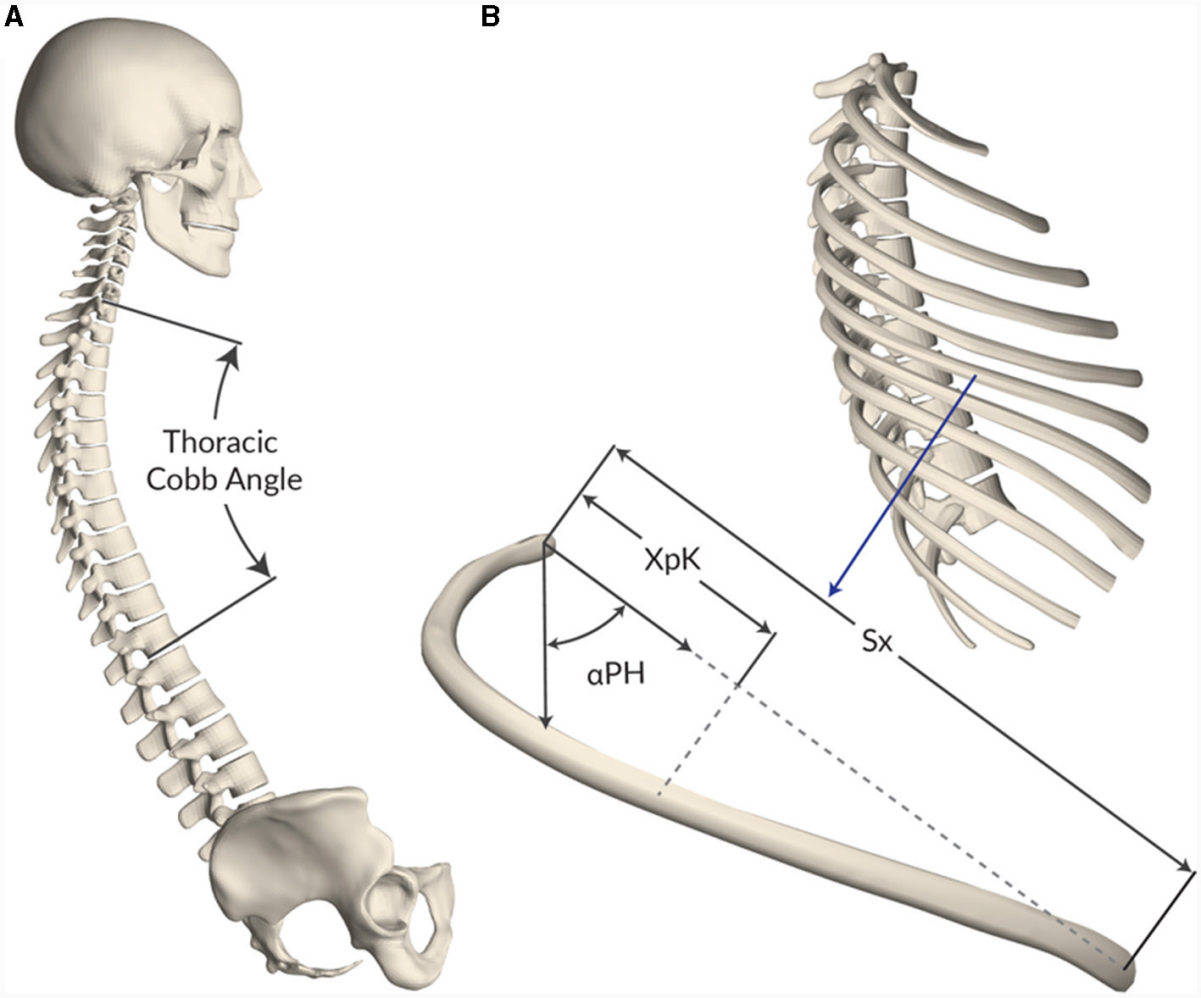
Schematic illustrating the measured geometrical features for the F05-O and F05-86G. **(A)** For the thoracic spine, the Cobb angle was measured and compared. **(B)** For the ribs, the rib angle (αPH), rib length (Sx) and location of the rib apex (Xpk) were measured and compare.

Since two geometries were evaluated in this study and with the objective of relating differences in injury outcome to geometrical features, both the F05-O and F05-86G were geometrically evaluated. Thoracic kyphosis and local rib morphology were measured and compared between geometries (F05-O and F05-86G). The rib morphology was measured using previously reported metrics and methods concerning rib angle (αPH), rib length (Sx), and location of the rib apex (Xpk) (Figure 3) (25). The measurements from the F05-O and the F05-86G were presented and compared.

### 2.3 Rib instrumentation for local rib kinetic assessment

**Figure 4:**
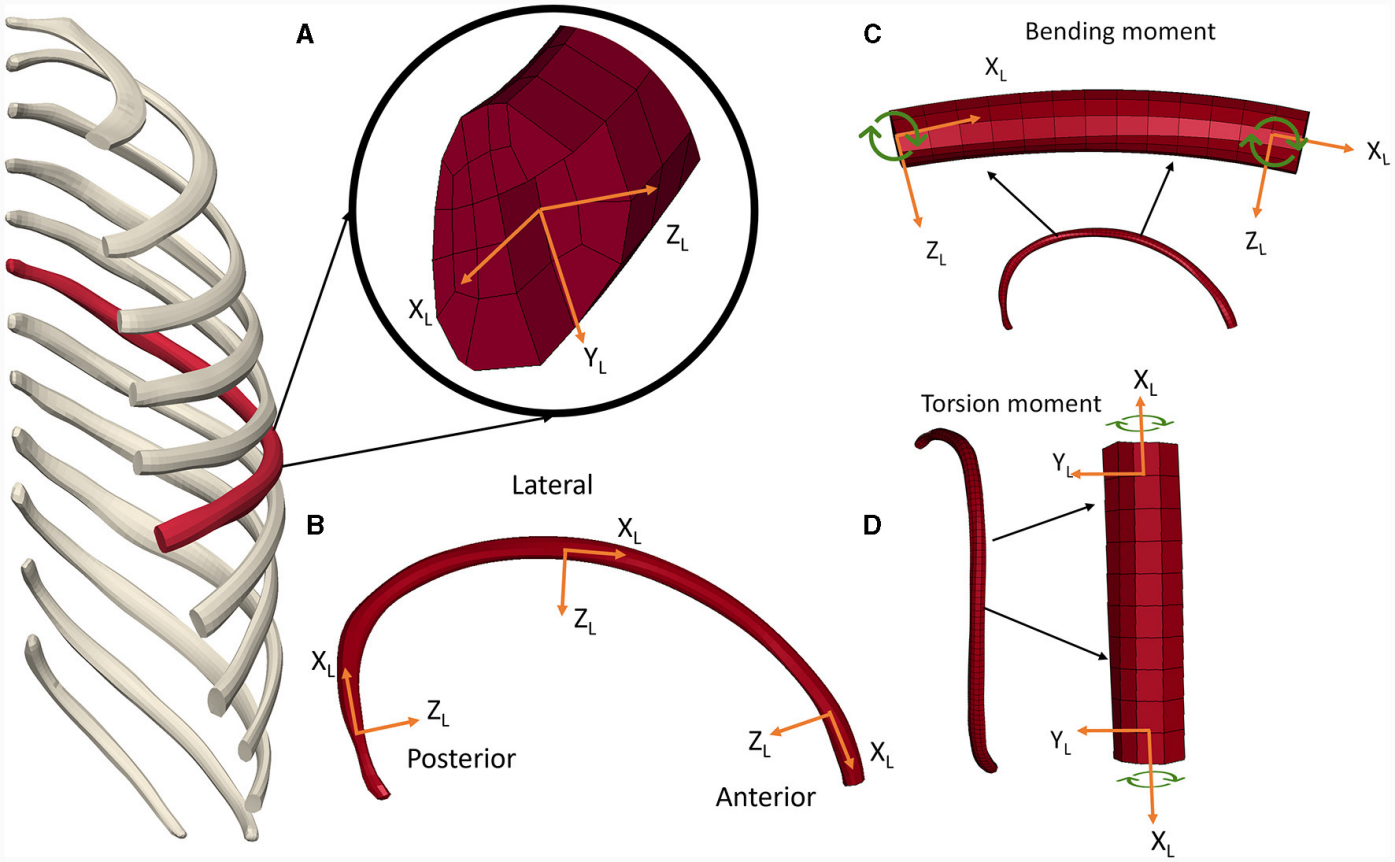
6^th^ rib instrumentation for rib kinetics calculation. **(A)** Local axis system definition in **(B)** posterior, lateral and anterior rib regions. **(C)** Bending moment and **(D)** torsional moment definition.

With the objective of assessing rib kinematic response throughout the rib length, the 6^th^ rib was instrumented in the anterior, lateral, and posterior regions. The rib kinetics were calculated in the local axis system where the “X_L_” axis orientation was defined by the rib length, the “Y_L_” axis by the primary axis of the rib cross-section, and the “Z_L_” axis defined by the cross product of “X_L_” and “Y_L_.” The “Z_L_” axis was generally aligned with the secondary axis of the rib cross-section (Figure 4). The origin was defined as the central node of the rib cross-section. The primary and secondary axis of the rib cross-section (Figure 4) were calculated in the anterior, lateral and posterior regions to define the local axis systems. The three forces and the three moments were extracted from the 6^th^ rib from the HBMs. The torsional moment (moment along X_L_), the bending moment (resultant moment of Z_L_ and Y_L_ moment), the normal force (force in the X_L_), and shear force (resultant force in the ZY_L_ plane) were calculated and reported.

### 2.4 Anatomically-positioned isolated rib simulations

**Figure 5:**
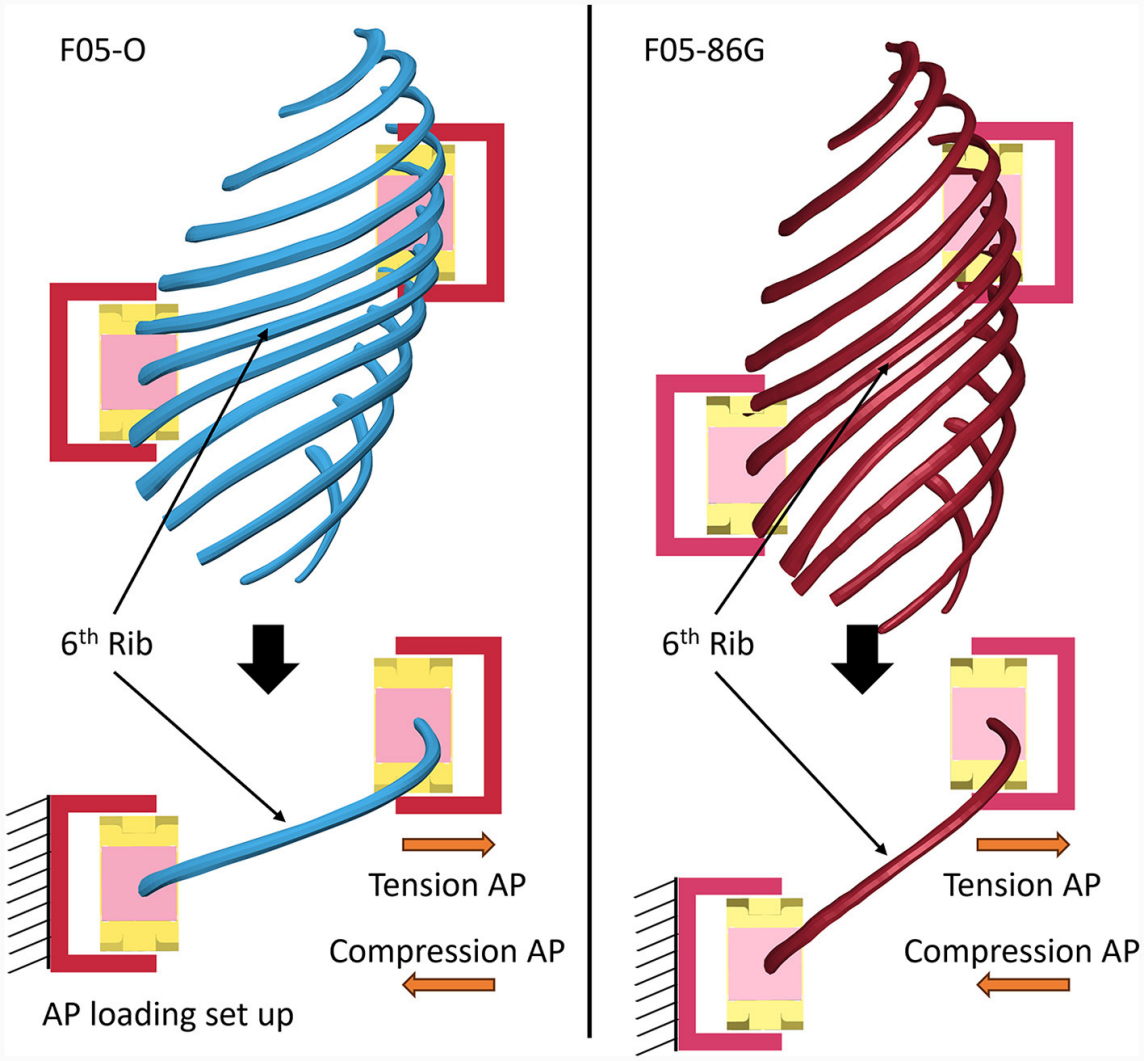
Definition of the Anterior-Posterior loading using the positioning of the full body for the 26-year-old geometry **(left)** and for the 86-year-old geometry **(right)**.

In a side impact, the mid-thorax (5^th^ to 7^th^ ribs) is primarily loaded, therefore, the 6^th^ rib was investigated in this study for the anatomically-positioned isolated rib simulations. In addition, previous studies (15, 16) have investigated the 6^th^ rib, allowing for direct comparison to the present study. The 6^th^ rib was extracted from the full body models and loaded in anterior-posterior loading in tension and compression (Figure 5). In a side impact condition, the rib span increases (tension) due to side thorax compression. In a frontal impact condition, the rib span decreases (compression) due to the frontal thorax compression. Therefore, the 6^th^ rib was loaded in AP compression and tension. The applied velocity-time curves were applied following previous experimental and computational studies (14, 16). The position of the ribs in the full body was maintained for the isolated rib simulations. The global force-displacement response, the local rib kinetic response in the anterior, lateral, and posterior regions and rib fracture location were evaluated and compared between the models.

### 2.5 Full-body side-impact simulations

The 26G and 86G models were settled in a previously published side impact sled model, representing the National Highway Traffic Safety Administration and Wayne State University designs (26, 27). The side impact sled model comprised a bench and set of rigid plates that impacted the body laterally. Ten side impact sled cases were analyzed; five HBMs (F05-O, F05_26_, F05_26GAmp_, F05_86_, F05_86Ymp_) loaded at two impact velocities. The impact velocities (3.3 m/s and 4.9 m/s) were applied to the sled while the HBMs remained unconstrained. Chest compression, rib kinetics and rib fracture were extracted from the models and compared between the models. Chest compression was defined as the change in distance between the most lateral regions of the left and right 6^th^ ribs. In this study, rib fracture was defined as complete transverse element erosion in the cortical and trabecular bone of the rib.

## 3 Results

### 3.1 Spine and local rib geometric assessment

**Figure 6:**
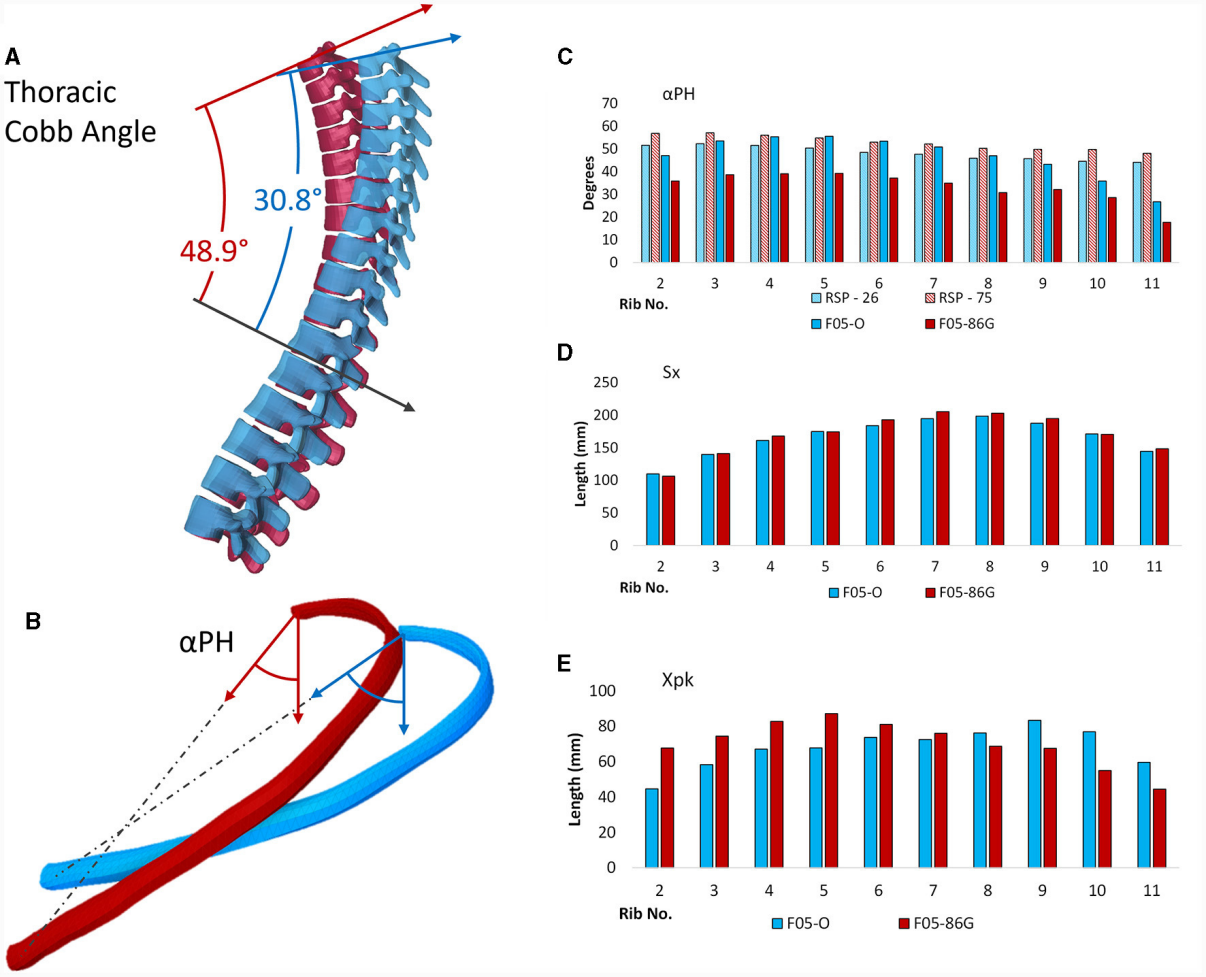
Rib geometry assessment: **(A)** Thoracic curvature characterized with Cobb angles, **(B)** definition of the rib angel (αPH), **(C)** rib angle for the models and population data (RSP, 27), **(D)** model rib length, and **(E)** rib apex location for the 26-year-old geometry (in blue) and 86-year-old geometry (in red).

The thoracic kyphosis of the F05-86G (48.9°) was higher when compared to that of the F05-O model (30.8°) (Figure 6A). Therefore, the rib angle (αPH) (Figure 6B) was lower (from the vertical axis) (Figure 6C) for all rib levels in the F05-86G when compared to the F05-O model. The rib length (Sx) (Figure 6C) was similar between both models. The rib apex location (Xpk) (Figure 6D) varied across the rib levels; in general, the upper rib levels (1^st^ to 7^th^ ribs) had a more anteriorly located apex in the F05-86G model when compared to the F05-O model and more posteriorly located in the lower levels (8^th^ to 12^th^ ribs) when compared to the F05-O model (Figure 6E).

### 3.2 Anatomically-positioned isolated rib simulations

**Figure 7:**
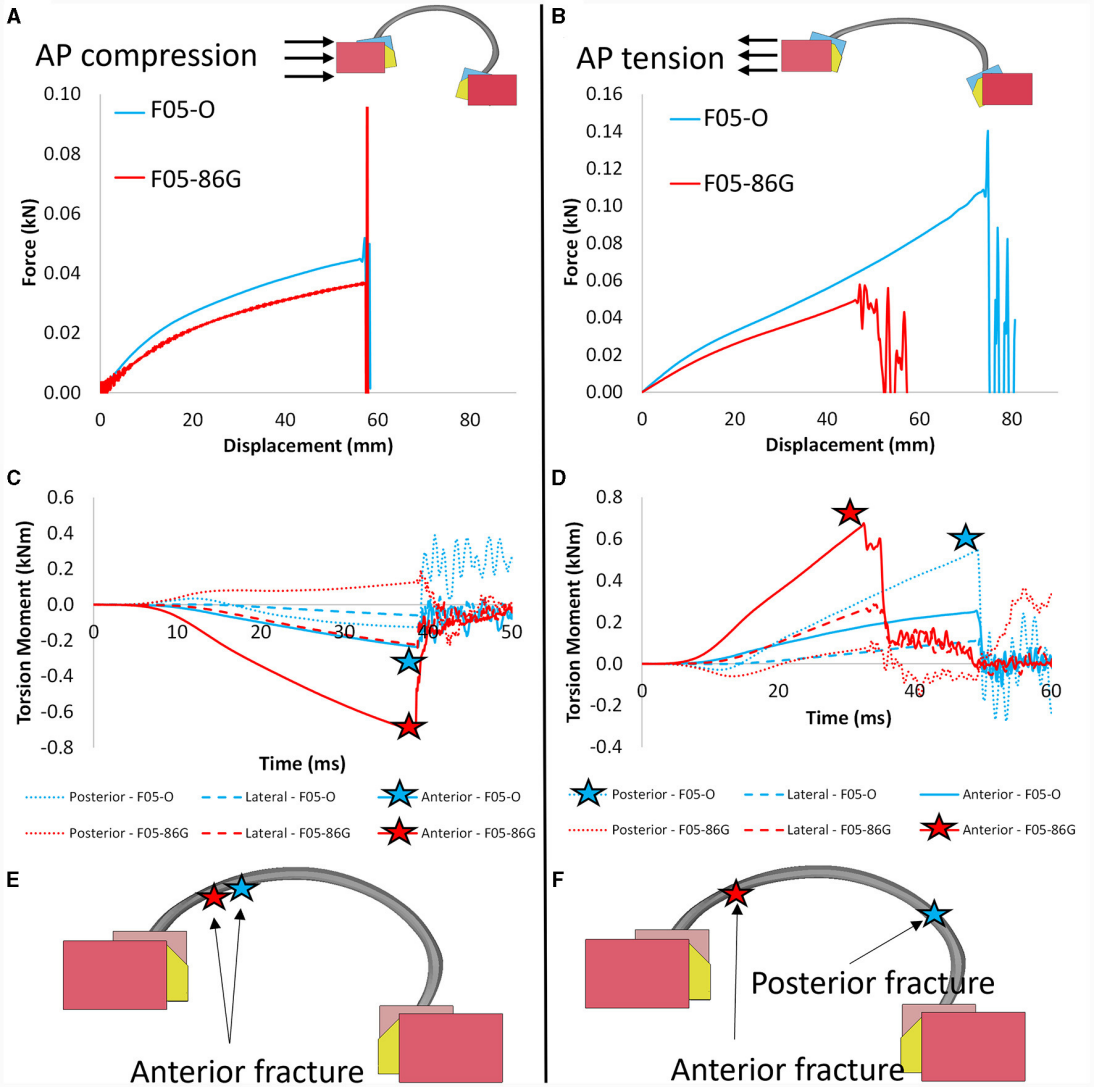
Force-displacement response for the 6^th^ rib in anterior-posterior **(A)** compression and **(B)** tension loading. Torsional moment in anterior-posterior **(C)** compression and **(D)** tension demonstrating higher moments for the F05-86G (red). Fracture location (bottom) for the F05-86G (red) and F05-O (blue) demonstrating changes in fracture location with increasing age in anterior-posterior **(E)** compression and **(F)** tension loading.

When isolating the geometric effect, in both AP compression and tension, the force to failure was lower for the aged geometry (Figures 7A, B). In AP tension, the displacement to failure was lower for the aged geometry (Figure 7A). In both compression and tension AP, the aged geometry demonstrated higher torsional moment in the location of the rib fracture (Figures 7C, D). In AP tension, the aged geometry affected the rib fracture location moving it from the posterior region in the young geometry to the anterior region in the aged geometry (Figure 7). The effect of aging material properties resulted in lower force and displacement to failure and lower torsional moment to failure maintaining the fracture location (Figure 7). The complete factorial analysis including the geometry and material properties effects on the isolated rib simulations can be found in the Appendix.

### 3.3 Full-body side impact simulations

**Figure 8:**
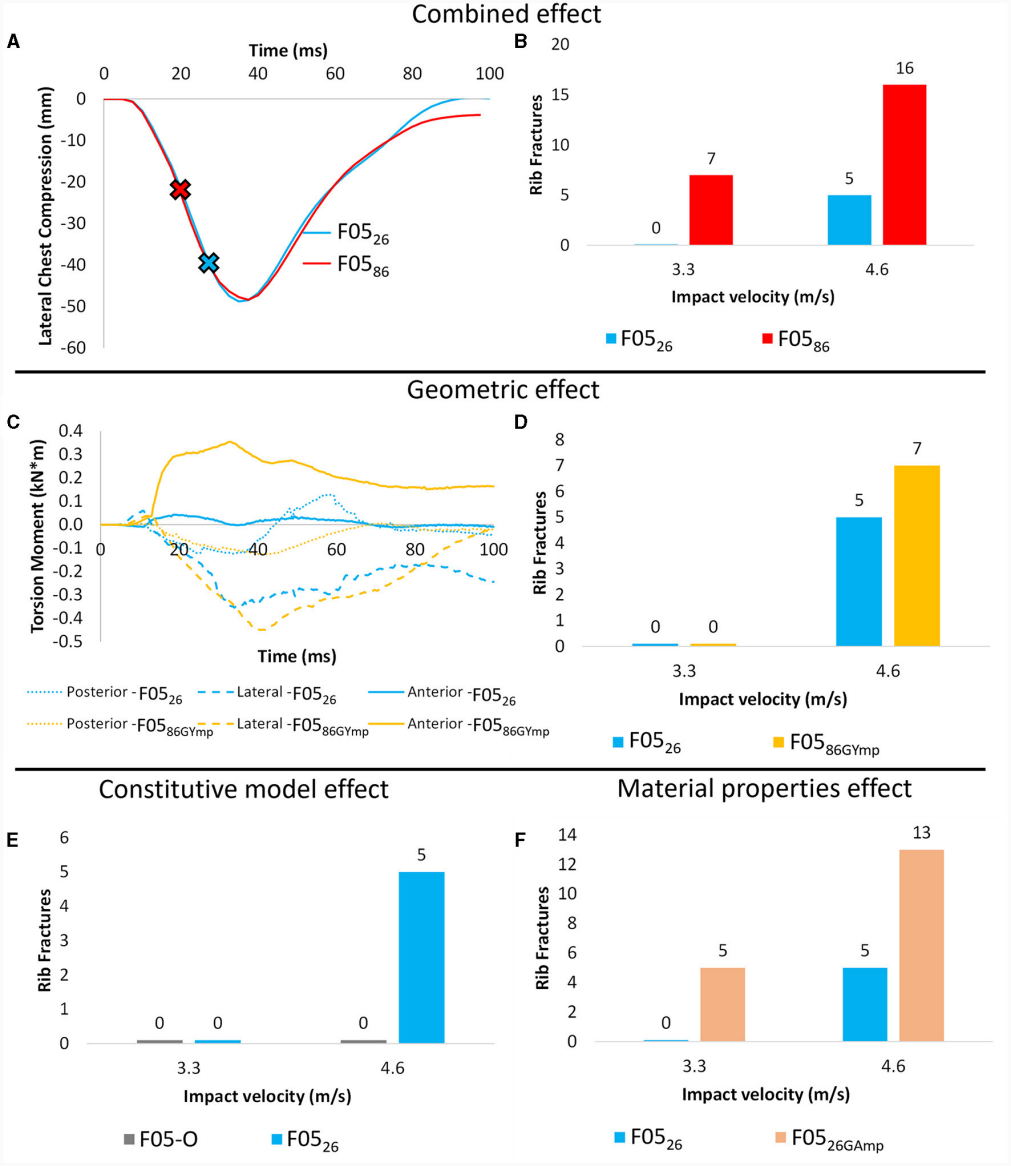
Combined effect of geometry and material properties on **(A)** chest compression in side impact at 4.6 m/s demonstrating the time at first rib fracture (fracture time indicated by “x”) and **(B)** rib fracture at 3.3 m/s and 4.6 m/s. Isolated effect of geometry on **(C)** 6^th^ rib torsion moment in a 3.3 m/s side impact (no fracture) and **(D)** on number of rib fractures. **(E)** Effect of including anisotropic material model with stress-triaxiality fracture criteria for the cortical bone model on number of rib fractures and **(F)** Isolated effect of age-adjusted material properties on rib fracture in side impact.

When assessing the models at the global level, both F05_26_ (combined young geometry and young material properties) and F05_86_ (combined aged geometry and aged material properties) models predicted similar lateral chest compression (Figure 8A). However, the rib fracture response varied between the young and aged models. The combined effect led to seven more rib fractures in the 3.3 m/s and eleven more rib fractures in the 4.6 m/s impact speed for the F05_86_ model when compared to the F05_26_ model (Figure 8B). Importantly, in the 6^th^ rib, the aged geometry led to higher torque in the anterior and posterior rib regions (Figure 8C). The isolated effect of the geometry on rib fracture was visible at the 4.6 m/s impact severity leading to two more rib fractures in the aged geometry model (Figure 8D). The isolated effect of aging the material properties on rib fracture was observed in both impact severities, with five and eight more rib fractures in the 3.3 and 4.6 m/s impact severity respectively (Figure 8F). The effect of enhancing the material models increased the number of rib fractures from zero to five in the 4.6 m/s impact severity (Figure 8E) while the effects were not apparent at lower impact severities. The factorial analysis results, including the geometry and material properties effects on the full body simulations, can be found in the Appendix.

## 4 Discussion

### 4.1 Geometry

Firstly, the subject-specific rib angles of the F05_86_ model were higher (further from the horizontal) than that of the F05_26_ model. In this study, the increased rib angle in the subject-specific aged model led to increased rib stresses and earlier failure of the ribs, with an increased number of rib fractures. It was noted that the rib angle in the subject-specific aged model was higher than the reported average for the population data (Figure 6C). The general trend in population data (28) indicates a small increase in rib angle with increasing age (Figure 6C). However, it has also been reported that population variability also increases with age (23). This study highlights the importance of subject-specific data, with the context of average population data, aiming to capture, in part, the increasing geometrical variability with increasing age and understand the effect of such variability on thorax injury. Although population values provide an overview of geometrical changes with age at the population level, the variability associated with rib angle for a given age may also help to explain the wide range of responses that have been reported in cadaveric studies for aged subjects.

The increased kyphosis in the subject-specific model resulted in a more forward-positioned rib relative to the population. The increased kyphosis coupled with the change in rib angles affected the local rib kinetics and ultimately the rib fracture. Previous studies focusing on geometric age effects on thoracic injury have focused on modifying the rib cage with T1 and T12 as anchor points and modestly modifying the rib angles, potentially missing the full geometric effect. During the morphing process (7), it was noted that the subject-specific rib angles were not achievable if the subject-specific thoracic posture was not considered, suggesting a strong relationship between posture and rib angles. The present study accounted for the changes in thoracic kyphosis coupled with the changes in rib angle, which had not been previously explored in earlier studies, offering a novel aspect to the investigation of thorax biomechanics.

### 4.2 Anatomically-positioned isolated rib simulations

A novel anatomically-positioned anterior-posterior isolated rib loading configuration was proposed. The modified rib orientation allowed for the examination of geometric changes and altered posture (i.e., increased thoracic kyphosis) with increasing age using a simplified boundary condition. The proposed configuration provides a middle-ground between existing anterior-posterior isolated rib loading set-up and full body experiments in terms of complexity. Importantly, it proposes a loading condition that captures the effect of *in-situ* rib position which is part of the human variability. This study proposed a loading condition that isolated the effect of *in-situ* rib position, that varies in the population. In this study, it was demonstrated that accounting for the anterior-posterior position and rib angle was critical to predict rib fracture.

At the isolated rib level, a strong relationship was found between rib angle further from the horizontal and higher torsional moment. The increased torsional moment led to higher stress levels in the cortical bone, which ultimately resulted in rib failure at lower force and displacement in the aged rib than in the younger rib. This particular geometric effect highlights the crucial role of the overall rib position (forward location in the aged geometry vs. the young geometry) and orientation (rib angle further from the horizontal) in determining the mechanical behavior of the rib structure under loading. Importantly, including an anisotropic material model with stress triaxiality fracture criteria in the models was critical to capture the geometric effect of aging included in this study. Previous research that has aimed to quantify the geometric effect of aging using a simplified material model might have underestimated the overall effect.

### 4.3 Full-body impact simulations

At the full body level, the investigation into the isolated effect of geometry demonstrated that higher torsional moments were induced due to the changes in rib angle and thoracic kyphosis, in agreement with the outcomes of isolated rib simulations. Moreover, the geometric effect also led to a higher number of rib fractures, suggesting an important role of geometry in determining the response of the aged population to impacts. In this study, exhaustive geometric aging of the model was carried out, encompassing overall posture, rib angles, and morphology leading to increased number of rib fractures. This comprehensive approach stands in contrast to previous studies that modestly account for aging in the geometry and suggested that the geometry played a modest role in the increased injury risk for the aged population.

Furthermore, the aging of material properties emerged as the primary factor influencing the number of rib fractures, with its impact being further amplified by the geometric factors included in this study. These findings emphasize the importance of considering both factors, material properties, and geometry, when investigating the biomechanical response of the aged population. Such integrated analyses are vital for a more accurate understanding of injury risk and response in older individuals, contributing to the improvement of safety measures and injury prevention strategies tailored to this specific demographic.

### 4.4 Limitations

First, only side impact was considered in the present study. Epidemiology indicates that frontal impact is an important condition to consider when evaluating chest injury. Future work will investigate the models in a frontal impact configuration.

This work included subject-specific geometries and population data for the material properties. To the author's knowledge, the relationship between age-related changes in geometry and material properties is unknown. Future research to understand the relationship, if any, between changing geometry and material properties with age.

In this study, cortical bone was investigated due to the structural relevance of the cortical bone being higher than that of the trabecular bone (29–31). It is acknowledged that trabecular bone properties and aging effects (6) can also contribute to rib response and will be investigated in future studies. Similarly, cortical bone thickness was not altered in the present study. Exploring the impact of cortical bone thickness changes associated with aging in future research may yield additional information on its role in age-related thorax injuries and fractures.

In this study, version 5.1 of the GHBMC F05-O detailed model was used. Although there have been improvements in other body regions in the more recent versions of the GHBMC model, this study focused on the ribs and the effect of rib angle, thoracic kyphosis, and rib cortical bone material properties on thorax response and injury. An advanced constitutive model for cortical bone was implemented, which contributed to explaining the difference in injury risk between young and aged subjects.

Lastly, the study did not consider changes in cross-sectional area due to aging. Addressing these areas of research in the future would contribute to a more comprehensive assessment of bone aging effects and their implications for the biomechanical response and injury risk in the aged population.

### 4.5 Conclusions

By incorporating biofidelic material models, the HBMs were able to demonstrate sensitivity to the combined effects of rib angle and age-adjusted material properties. The current study highlights the importance of using biofidelic material models that accurately represent the material response in parametric studies. Using simplified material models (e.g., isotropic) and fracture criteria (e.g., metals plasticity) could overlook important factors in response and injury risk.

Accounting for age-related changes in overall posture, including thorax curvature, rib angles and rib morphology, proved essential in predicting higher rib fractures for the aged models. Increasing rib angle from horizontal, particularly in the aged model, led to higher rib torsional moments (and higher stress in the cortical bone), contributing to the increased number of rib fractures observed in the aged model. Importantly, the subject-specific rib angles were only achievable if the subject-specific thoracic curvature was considered. The present findings suggest that, considering overall posture and rib angles when studying age-related thoracic biomechanics is important, as these factors are coupled and play a critical role in the injury outcome.

The loading of a single rib in anterior-posterior (AP) loading, maintaining the anatomical rib angle, isolates the geometric factors considered in this study in a simplified load case. The present study showed that the position in the rib, relative to the loading (e.g., rib angle), played a strong role in the rib mechanical response. In particular, the isolated rib simulations demonstrated the importance of torsion in leading to earlier rib fracture, generated by offset loads due to rib angle. The proposed simplified loading set-up can be used in future work aimed at investigating rib mechanics accounting for the rib body positioning.

The present study showed that the total number of rib fractures, time at fracture, and rib local kinetics have a high sensitivity to rib angle and age-adjusted cortical bone material properties. Higher rib angles (further from the horizontal) led to higher stresses, including torsional stresses on the rib for the aged model, demonstrating an important difference between the young and aged models. In contrast, chest compression demonstrated no change between the young and aged models investigated. The lack of sensitivity may be due to the measured chest deflection including local rib deflection as well as gross chest deformation, and the location of the measurement points. The outcome of this study suggests that other metrics outside of chest compression may be necessary when assessing injury risk for aged people.

## Data availability statement

The original contributions presented in the study are included in the article/Supplementary material, further inquiries can be directed to the corresponding author.

## Author contributions

MC: Conceptualization, Data curation, Formal analysis, Investigation, Methodology, Software, Validation, Visualization, Writing—original draft, Writing—review & editing. JB: Funding acquisition, Resources, Supervision, Writing—review & editing. BP: Funding acquisition, Resources, Supervision, Writing—review & editing. CM: Funding acquisition, Resources, Supervision, Writing—review & editing. DC: Conceptualization, Data curation, Formal analysis, Funding acquisition, Investigation, Methodology, Project administration, Resources, Software, Supervision, Validation, Visualization, Writing—review & editing.
