# Supplementary material for: Explaining and predicting the increased thorax injury in aged females: age and subject-specific thorax geometry coupled with improved bone constitutive models and age-specific material properties evaluated in side impact conditions
Source: Front Public Health. 2024 Mar 11;12:1336518. doi: 10.3389/fpubh.2024.1336518 (PMC10964717; doi:10.3389/fpubh.2024.1336518)
Supplement: Supplementary file 1 [file Data_Sheet_1.docx]

Appendix

# Factorial Analysis of the Physiological Single Rib Simulations

Factorial analysis including the geometry and material properties effects on the isolated rib simulations (Figure A1).

*Figure A1: Force-displacement and torsion moment response for the 6th rib in physiological anterior-posterior compression (left) and tension (right) loading demonstrating the isolated effect of the age-adjusted material properties and geometry. Blue and red denotes young and aged geometry respectively and solid and dashed lines denotes young and aged material properties respectively.*

# Factorial Analysis of the Full Body Side Impact Simulations

factorial analysis including the geometry and material properties effects on the full body simulations (Figure A2) showing the isolated effect of the age-adjusted material properties and geometry.

Figure A2: Isolated effect of age-adjusted material properties on rib fracture in side impact. Blue and red denote young and aged geometry respectively and solid and dashed bars denote young and aged material properties respectively.
